# Automatic tree parameter extraction by a Mobile LiDAR System in an urban context

**DOI:** 10.1371/journal.pone.0196004

**Published:** 2018-04-24

**Authors:** Mónica Herrero-Huerta, Roderik Lindenbergh, Pablo Rodríguez-Gonzálvez

**Affiliations:** 1 Department of Geoscience and Remote Sensing, Delft University of Technology, Delft, The Netherlands; 2 TIDOP Research Group, Higher Polytechnic School of Avila, University of Salamanca, Avila, Spain; 3 Department of Agronomy, Purdue University, West-Lafayette, Indiana, United States of America; 4 Department of Mining Technology, Topography and Structures, Universidad de León, Ponferrada, Spain; University of Siena, ITALY

## Abstract

In an urban context, tree data are used in city planning, in locating hazardous trees and in environmental monitoring. This study focuses on developing an innovative methodology to automatically estimate the most relevant individual structural parameters of urban trees sampled by a Mobile LiDAR System at city level. These parameters include the Diameter at Breast Height (DBH), which was estimated by circle fitting of the points belonging to different height bins using RANSAC. In the case of non-circular trees, DBH is calculated by the maximum distance between extreme points. Tree sizes were extracted through a connectivity analysis. Crown Base Height, defined as the length until the bottom of the live crown, was calculated by voxelization techniques. For estimating Canopy Volume, procedures of mesh generation and α-shape methods were implemented. Also, tree location coordinates were obtained by means of Principal Component Analysis. The workflow has been validated on 29 trees of different species sampling a stretch of road 750 m long in Delft (The Netherlands) and tested on a larger dataset containing 58 individual trees. The validation was done against field measurements. DBH parameter had a correlation R^2^ value of 0.92 for the height bin of 20 cm which provided the best results. Moreover, the influence of the number of points used for DBH estimation, considering different height bins, was investigated. The assessment of the other inventory parameters yield correlation coefficients higher than 0.91. The quality of the results confirms the feasibility of the proposed methodology, providing scalability to a comprehensive analysis of urban trees.

## Introduction

Tree inventory information and monitoring tree changes over time are critical input for tree management systems, ecosystem services and aboveground biomass estimation. This information could also show the ecosystem influence on climate change, carbon and water cycling [1]. Furthermore, the canopy structure affects the radiation regime and other biochemical and ecological processes. In an urban context, tree register data is used in city and environmental planning, in deriving and modelling urban pollution and temperatures, in locating hazardous trees and for biodiversity monitoring [2]. Recently, the municipality of Delft (The Netherlands) has opened a data-base where all the trees from the urban area of Delft are listed with properties as location, DBH range, dimension range, species health conditions, year of plantation and inspection date [3].

Dendometric variables have traditionally been estimated with field campaigns. The advent of recent remote and near sensing technologies has opened up a new field of possibilities for carrying out such work through non-destructive methods, providing advantages regarding the economic costs involved, the time invested and the quality of results [4]. In that sense, the use of active high-resolution sensors, such as Light Detection and Ranging (LiDAR) enables high accuracy in estimation of tree parameters. This active remote sensing technology based on the principle of laser ranging [5], provides precise and efficient information on the horizontal and vertical distribution of vegetation and canopy structure. Many studies also use other data sources, such as digital aerial photographs [6] or combine high resolution and hyperspectral images with LiDAR data in urban vegetation mapping [7]. A comprehensive review of the application of LiDAR remote sensing for the retrieval of forest structural parameters at different scales is provided by [8].

More recently, Mobile LiDAR Systems (MLS) have emerged as an excellent tool to assess urban structural tree parameters and the distribution of their constituents, enabling fast and accurately capturing of 3D data of individual trees with high spatial detail along the road [9]. However, the size of the data files can be considerable, complicating the handling and storage of 3D information and requiring long processing times. MLS operates at a scale between manual and airborne LiDAR measurements and has better acquisition geometry for trees. Indeed, airborne LiDAR has a limited ability to sample the vertical distribution of the canopy [10] by the narrow near nadir scan angle and density of vegetation causing occlusions. Since a MLS involves several sensors to acquire a georeferenced 3D point cloud, the final precision includes individual error sources, like LiDAR, Inertial measurement unit (IMU) and global navigation satellite systems (GNSS). Comparisons proved that an accuracy better than 3 cm (standard deviation of the differences between measured and reference data) can be achieved by the system in good GNSS conditions [11]. The main disadvantage of MLS of having no-access to non-transitable areas is being dismissed by the new developments of MLS boarded in a backpack [12]. However these solutions are still being tested in terms of precision and performance in different scenarios. Another related technology is Terrestrial Laser Scanner (TLS), which has been employed for mapping vegetation properties and tree reconstruction [13]. But it is time-consuming compared with MLS in an urban context. Henning et al. [14] applied a TLS in a study area of 20x40 m^2^ in a species-limited and non-urban context. However, the completeness of the tree representation might be less for MLS compared to TLS campaigns, where trees are typically scanned from multiple positions.

Diameter at Breast Height (DBH) is considered an essential parameter in tree allometry. Many countries store the DBH of urban trees in cadaster databases for monitoring purposes [3]. Several studies estimated DBH from TLS [15] and airborne LiDAR [16]. MLS data has already been used to estimate stem diameter in a forest environment from the intensity captured by the laser from different echoes, reaching a RMSE of 14% [17]. Other critical measurements are Tree Height (TH) and Base Crown Volume (BCV) that afford qualitative information about the stand and quantitative tree information. Correlation with *in situ* data with TLS based DBH and TH were reported to be between 0.91 and 0.97 and 0.92, respectively [15]. The tree stem curve could help to describe the BCV, specifying the stem tapering as a function of the height. Another key parameter is the Crown Base Height (CBH) normally used in fire modelling, which can be estimated from airborne LiDAR data through voxelization techniques based on moving voxels [18]. However, estimating CBH is a challenging task because, normally, it is difficult to measure it in the field. A novel method to obtain these tree parameters from MLS data is developed by [19] using voxelization techniques reaching a R^2^ value of over 0.8.

**Fig 1:**
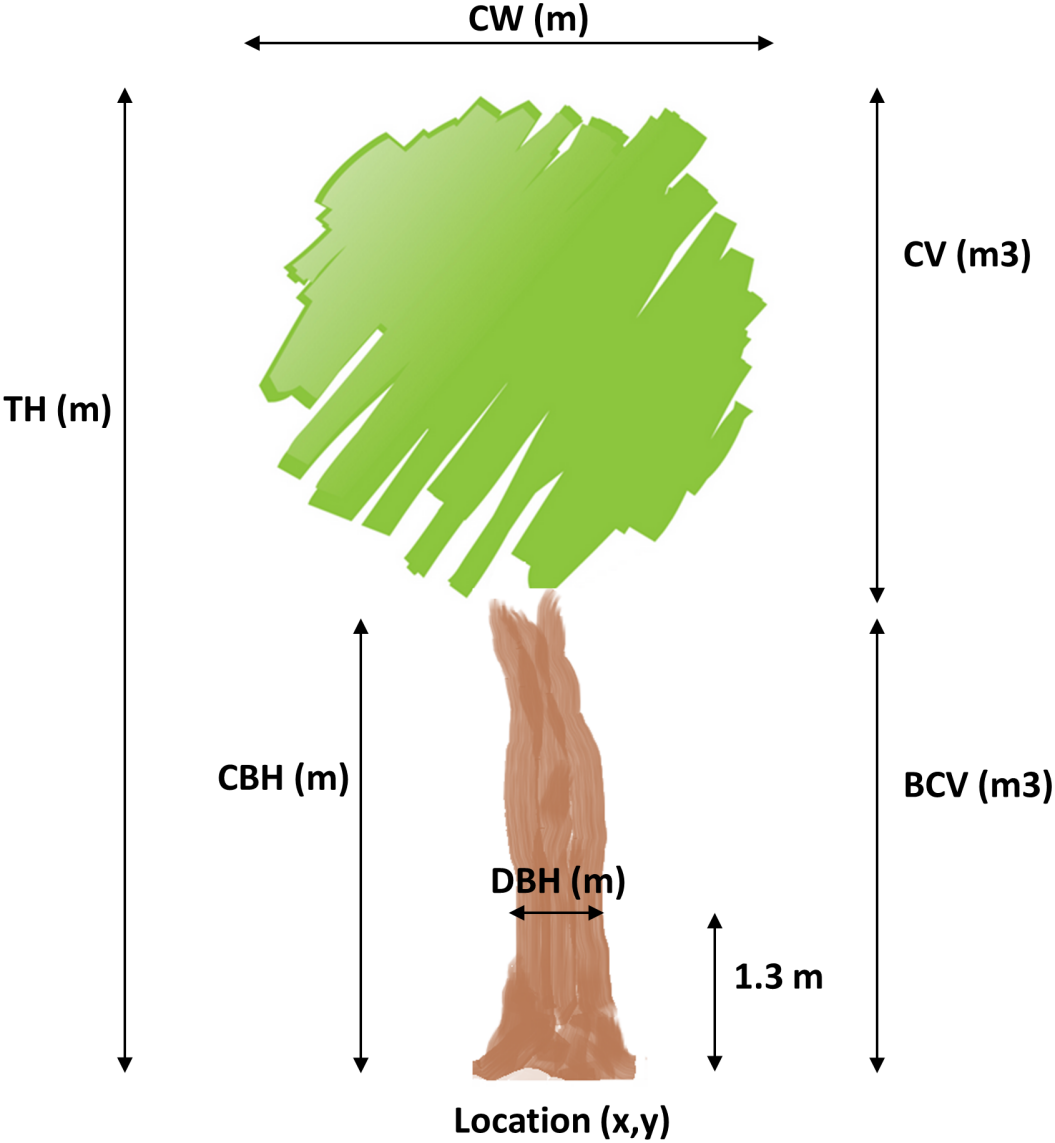
Schema of tree parameters. Location coordinates (x, y), Tree Height (TH), Crown Width (CW), Diameter at Breast Height (DBH), Crown Base Height (CBH), Base Crown Volume (BCV) and Canopy Volume (CV).

The accurate measurement of canopy architecture and tree crown parameters such as Crown Width (CW) or Canopy Volume (CV) is critical for plant physiology and vegetal health monitoring, typically related to photosynthesis and energy transfer. To date, little research has been done to retrieve Crown Width using TLS [20] and the estimations with airborne LiDAR reach coefficients of determination as low as 0.63 [7]. Moreover, several studies have been focused on modelling the canopy to estimate tree crown variables using TLS and airborne LiDAR [21]. Fig 1 represents a schematic cartoon of the mentioned tree parameters.

The aim of the present work is to develop an efficient and precise methodology to obtain structural parameters of individual trees in urban inventories at city level based on point clouds derived from MLS. For the extraction of individual trees, the pipeline shown and tested in [22] at large scales is employed. However this initial approach only estimated tree size and location. The methodology proposed here has to face difficulties due to the variable driving speed (different point density) and the specific acquisition geometry of the MLS technology, resulting in partial 3D data (only one side view), and presence of noise in the data. Occluding vegetation may lead to underestimation of selected parameters compared to manually collected field data [23]. In this work, the following parameters are estimated: location coordinates, Tree Height (TH), Crown Width (CW), Diameter at Breast Height (DBH), Crown Base Height (CBH), Base Crown Volume (BCV) and Canopy Volume (CV). More concretely, a connectivity analysis is performed to calculate TH and CW; voxelization technique is employed to estimate CBH. From these parameters, the stem curve is computed until CBH. A RANSAC-based fitting in conjunction with a dimensional analysis of the variance covariance matrix by Principal Component Analysis (PCA) is used to estimate tree location, tree orientation and DBH. CV is obtained by canopy meshing followed by an α-shape refinement step. To validate the methodology, field measurements were taken from 29 trees of different species. Furthermore, this workflow was tested on an additional dataset which contains 58 trees in order to provide scalability. Additionally, the influence of the number of points from different height bins on the accuracy of DBH estimations was investigated.

The proposed methodology is part of a robust and efficient workflow which considers the automated extraction of tree parameters, and which has been implemented in the infrastructure of the FP7 IQmulus project, as part of the so-called urban showcase [24].

## Materials and methods

### Acquisition system

**Fig 2:**
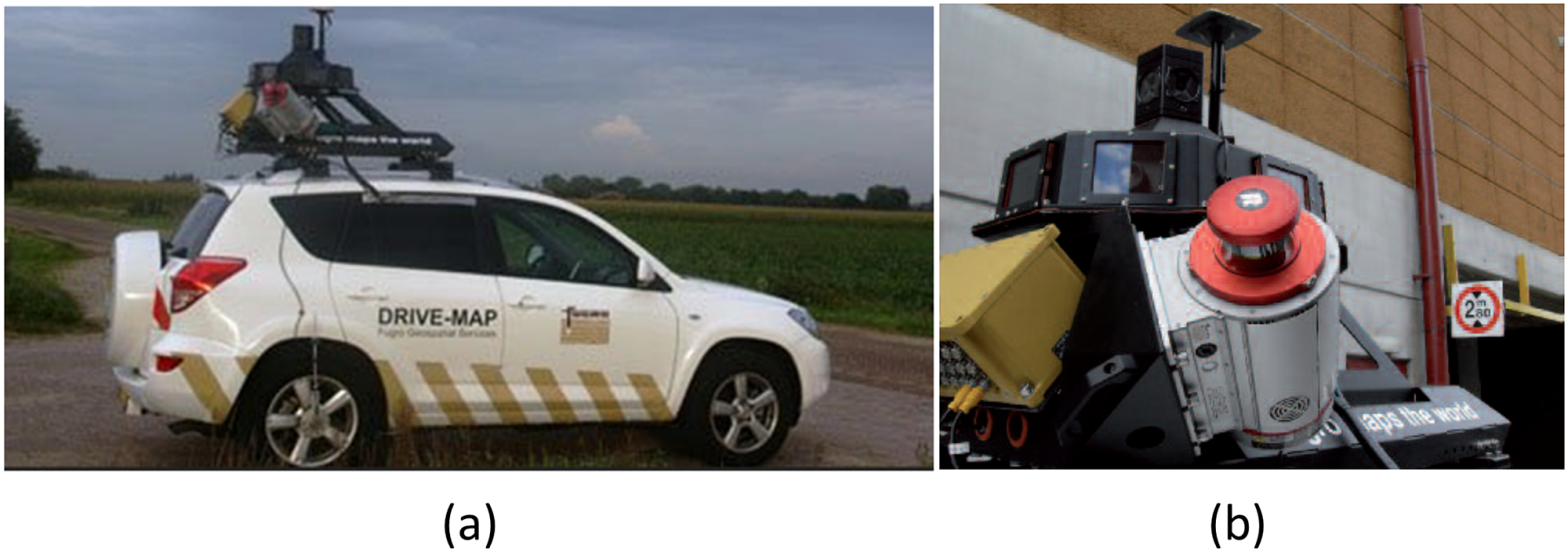
Fugro Drive-map system used for the data acquisition (a) and its LiDAR System (b) (source: Fugro).

The acquisition system used in this work is the Fugro Drivemap system, shown in Fig 2. This MLS is composed of two high performance Riegl VQ250 laser scanners, an all-terrain vehicle and a navigation system. The navigation system of the vehicle is the Applanix POS LV 520 that integrates a GNSS receiver, an IMU and a distance measuring instrument. The IMU defines the centre of the vehicle coordinate system and, accordingly, the other sensors were aligned with it. The GNSS positioning system is used for mapping vehicle local coordinates to absolute coordinates with a planimetric precision of about 0.02 m. It should be noted that precision properties are highly dependent on GNSS availability, which can be considered good for this study. The GNSS-receiver has 10 Hz positioning rate and the laser technical specifications are defined in Table 1.

**Table 1:** Technical specifications of the Mobile LiDAR System.

Parameter	Value
Laser Pulse Rate	0.133·10^6^ pulses / s
Laser Point Density	115000 points / m^2^
Field of View	360^0^ * 0.0023^0^
Ranging Accuracy	< 2 cm
Sweep Angle	360^0^
Maximum Range	30 m (0.10 reflectivity)

### Methodology for estimating structural tree parameters

**Fig 3:**
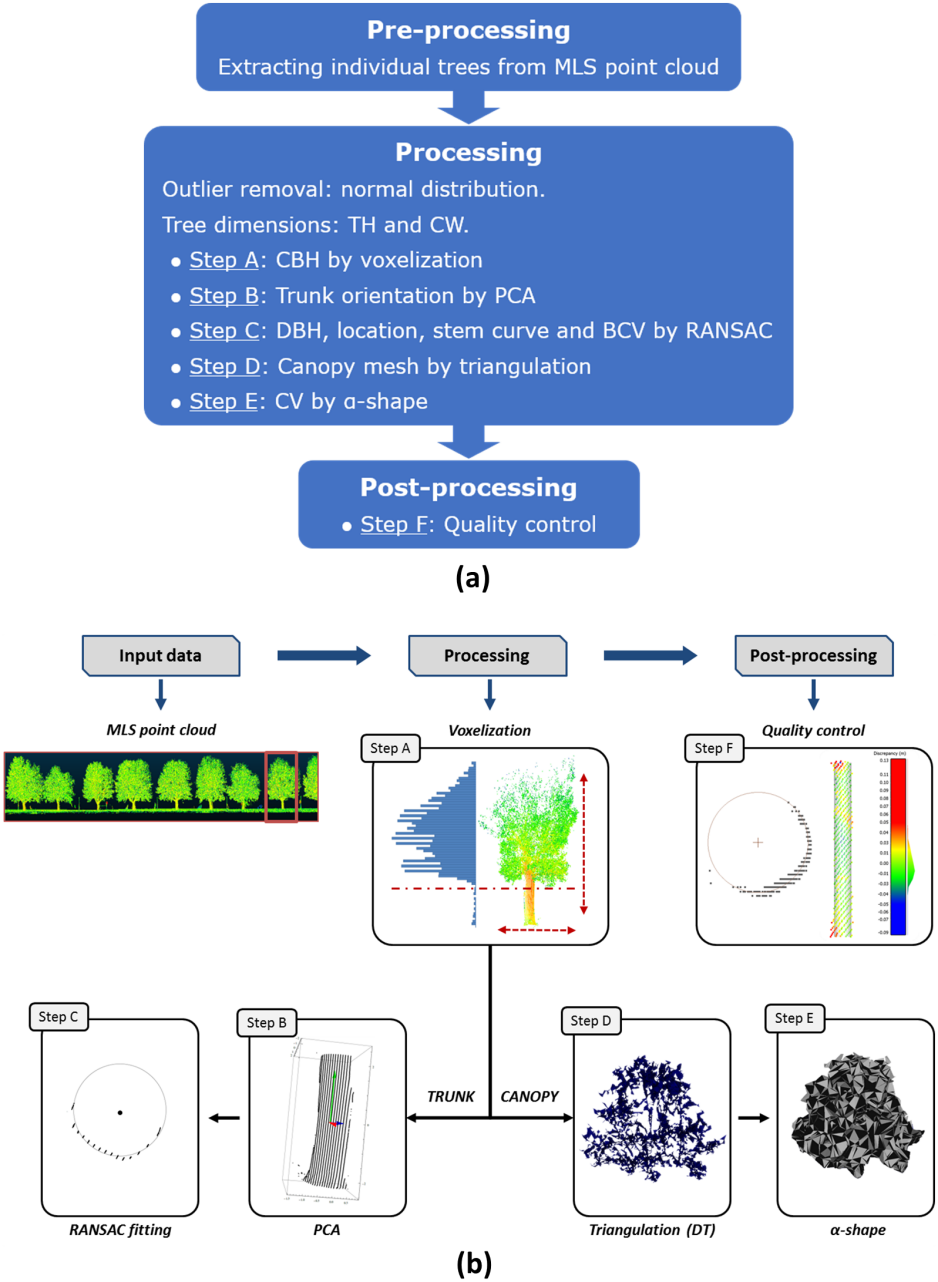
Workflow of the methodology for structural tree parameter extraction; scheme (Fig 3a) and illustrated workflow (Fig 3b).

The proposed methodology aims at extracting structural tree parameters derived from MLS data. Before estimating these parameters, the extraction of individual trees from the point cloud is necessary through an automated workflow by voxel analysis, as explained in [25]. Assuming a point cloud consists of a number of separated trees, the following steps are performed for each individual tree, as Fig 3 illustrates.

The proposed methodology was implemented in C++ using Point Cloud Library (PCL) [26] and compiled and run on the Ubuntu 14.04 64-bit operating system. The computational cost of the algorithm regarding the run time can be defined as a real time process. For the α-shape calculations, the open-source 3D mesh processing system is used [27].

#### Outlier removal

The point cloud sample may contain outliers and noisy points caused by interference effects (persons, vehicles,…). Such points are not regarded as samples of actual trees and the first step is to filter them from the point cloud. To remove isolated points or small point clusters, a statistical analysis on each point’s neighbourhood is performed by assuming a Gaussian distribution of neighbours’ distances (*d*). This distances are weighting with the variable point densities due to the combined effect of the distance to the sensor, occlusions and driving speed. So, for each MLS point, the average distance to its *k*-nearest neighbours are computed and evaluated against the Upper Confidence Limit (UCL) of a normal error distribution. A *j*-th point (*j* = 1…*k*) will be excluded as outlier if the ratio between their distance and the UCL, for a given confidence level (*p*), is higher than 1, as is shown in (1).

$$threshold=\frac{d_{j}}{\bar{d}+p\cdot \sigma_{d}}>1$$ (1)

where $\bar{d}$ and σ_d_ are the mean and standard deviation of the distances to the *k*-nearest neighbours in the evaluated point vicinity (*j* = 1…*k*) respectively. The confidence level (*p*) is expressed as the critical value associated in the standard normal density curve.

#### Tree Height (TH) and Crown Width (CW)

Starting from the point clouds of each individual tree, Tree Height and Crown Width are estimated by extracting the minimal and maximal points in the three Cartesian coordinate directions from the MLS dataset, taking into account the point cloud density and their connectivity [28]. These extreme points should be computed from a minimum sample size (spatial resolution) and the distance between the closest voxels should be less than a predefined threshold (spatial connectivity).

#### Crown Base Height (CBH)

For this parameter, individual trees are uniformly downsampled through a 3D voxel data structure (Fig 3, step A).

The voxels are defined by the side of the cube, which is directly related to the a-priori spatial resolution of the MLS data. As results, the originally gathered points are substituted by the coordinates of the voxel center defined by the mean of the points in the voxel generating a new 3D model space. The advantages of using voxels is that their use reduces the computational time of the subsequent steps and decreases the effects of tree branches.

The tree vertical distribution is characterized by the voxel distribution along the Z axis (since trees are vertically oriented). For each vertical interval, the number of LiDAR hits is accumulated (Fig 3, step A). As result, a histogram of the vertical distribution is generated [29]. The CBH is defined as the lowest inflexion point of the histogram (the inflexion of a curve is a point where the curvature changes its sign while a tangent exists) under the null hypothesis that the lowest living branch belongs to the crown. As a result, the trunk can be separated from the canopy using the estimated CBH value.

#### Trunk orientation by PCA

In MLS data, trunks are not necessarily orthogonal to the XY plane, as many trees have a more or less tilted trunk. In order to assess the trunk direction, the trunk 3D points bounded by the ground and the CBH (Fig 3, step B) are evaluated by a PCA algorithm [30]. Hereafter, the 3D voxel data structure will not be used anymore, but we return to the original point cloud data. This statistical analysis uses the first and second moments of these points and results in three orthogonal vectors centred on the centroid of the point cloud. The PCA synthesizes the distribution of points along the three dimensions, and therefore models the principal directions and magnitudes of variation of the point distribution around the centre of gravity.

Considering a 3D point cloud with *k* points sampling the trunk surface, the coordinates *xi*, *y*_*i*_ and *z*_*i*_ for each point *i = 1*,… *k* are collected in a matrix *X* (2).

$$\text{X}=\left(\begin{array}{ccc} x_{1} & y_{1} & z_{1}\\ \vdots & \vdots & \vdots \\ x_{k} & y_{k} & z_{k} \end{array}\right)=\left(\begin{array}{c} {\vec{x}}_{1}\\ \vdots \\ {\vec{x}}_{k} \end{array}\right)$$ (2)

The covariance matrix (Σ) of the trunk point cloud (X) is defined by:

$$\sum =\frac{X^{T}X}{\text{k}}=\left(\begin{array}{ccc} \sigma_{x}^{2} & \sigma_{xy} & \sigma_{xz}\\ \sigma_{xy} & \sigma_{y}^{2} & \sigma_{yz}\\ \sigma_{xz} & \sigma_{yz} & \sigma_{z}^{2} \end{array}\right)$$ (3)

$$\sigma_{x}^{2}=E\left(x^{2}\right)-E{\left(x\right)}^{2}=\sum_{i=1}^{k}{\left(x_{i}-\bar{x}\right)}^{2}$$ (4)

$$\sigma_{xy}^{}=E\left(xy\right)-E\left(x\right)E\left(y\right)=\sum_{i=1}^{k}\left(y_{i}-\bar{y}\right)\left(x_{i}-\bar{x}\right)$$ (5)

where *E(j)* is the expected value of the *j*-th dimension, (*σ*^2^_*x*_, *σ*^2^_*y*_, *σ*^2^_*z*_) are the variances of the variables, and the elements outside the main diagonal of Σ are the covariances for each pair of variables. The principal trunk direction is defined as the first eigenvector of the covariance matrix.

#### DBH and tree location through RANSAC

Due to possible variation of spatial resolution of the MLS points, different bins or intervals are considered to achieve a robust estimation of the DBH parameter. More concretely, with respect to the ground level, Z = 0, trunk points have been extracted from the following vertical bin intervals: (a) 1.25–1.35 m; (b) 1.20–1.40 m; (c) 1.10–1.50 m and (d) 1.00–1.60 m. The selected points are projected into a plane orthogonal to the axis corresponding to the principal direction of the trunk (see previous step), under the hypothesis that the diameter does not vary significantly along a short length of the stem for mature trees.

DBH is estimated by means of RANSAC (RANdom SAmple Consensus) circle fitting. This estimator has favourable properties such as robustness, generality and simplicity. RANSAC extracts arbitrary primitive types by randomly drawing minimal sets from the point data and is reliable even in the presence of a considerable proportion of outliers [31]. In an iterative process, a minimal set of points is randomly selected to define the geometric primitive, which is evaluated against the sample. The number of required iterations depends on the probability to find an outlier free sample. The thereshold that defines if a point is considered an inlier is based on the a-priori MLS error, as specified by the MLS manufacturer. Circle fitting is applied to the reference XY plane defined by the PCA approach to estimate DBH (Fig 3, step C). This procedure fits a partially sampled circle. First, points from the selected DBH bin are projected to the reference xy plane, obtained for the PCA approach. The projected points outline a partially sampled circle. Next, the tree location is defined as the projection of the derived circle centre in the trunk’s principal direction by its intersecting with the terrain. The diameter of this circle is estimated using RANSAC. The diameter is an estimator of the DBH.

#### Stem curve and Base Crown Volume (BCV)

The stem curve is automatically derived from the point cloud, between the minimum height and CBH starting from diameters at 0.65 meter above the ground, followed by diameters at the DBH height and consecutively at every meter above the DBH height until the CBH is reached. To determine the diameter at different heights, the RANSAC circle fitting approach is followed, estimating circle parameters of the projected trunk points at 0.20 m interval size. As a constraint, the diameter is required to remain constant or decrease with increasing height.

The BCV (dm^3^) is computed from the stem curve, summing the volume of each stem section, as shown in (6):

$$BCV=\sum_{i=0}^{m}v_{i}=\frac{\pi }{40}D_{1}^{2}\cdot 0.65+\sum_{i=1}^{m}\frac{\pi }{160}{\left(D_{i}+D_{i+1}\right)}^{2}\cdot l_{i}$$ (6)

where *m* is the number of stem sections, *v*_*i*_ is the volume of the *i-th* stem section in dm^3^, *D*_*i*_ is the diameter of the end of the *i-th* section in cm, and *l*_*i*_ is the length of the *i-th* stem section in m. Eq (6) assumes that the cross-section of the tree stem is circular. In fact, tree stem shapes exhibit an abundant variety since different tree species have diverse forms and irregularities such as knots and bulges. However the circular shape is most often employed to generalize the stem sections when the tree trunk is modelled [32].

#### Canopy Volume (CV) by generating a canopy mesh

The foliage pattern elements are important for understanding the radiation regime and canopy structure. LiDAR systems acquire detailed measurements corresponding to the 3D distribution of canopy components [33]. However, the acquisition is only partial due to the one-sided field of view of the MLS. This subsection is devoted to synthesize and quantify the canopy shape (canopy volume—CV) from the MLS partial point clouds of the individual trees.

The approach is supported by 3D modelling of the point cloud of the canopy by a 3D Delaunay triangulation (DT) [34]. The result is a triangulated irregular network (TIN) which requires a filter phase to obtain a tree canopy model close to reality. The approximation shown in [35] is used in this step which incorporates several automatic and sequential tasks:

- Hole filling based on interpolators of radial basis function [36].
- Fill meshing gaps, based on minimum threshold distance algorithms.
- Removal of topological noise, allowing the mesh to be re-triangulated locally.
- Removal of geometric noise by algorithms that apply filters such as anti-aliased Laplacians in general or in specific zones [37].

Once the canopy mesh is reconstructed (Fig 3, step D), an approximate envelop of the mesh is computed using α-shape techniques [38]. The intermediate step of calculating the canopy mesh, improves the efficiency of the α-shape processing of the dense point cloud of the canopy.

An α-shape is a family of piecewise parametric simple curves in Euclidean space associated with a set of points. The α-shape of a finite point set is a polytope that is uniquely determined by the set and a real number α ϵ [0, ∞], being in the extreme case (α = ∞) coincident with the convex hull. As the real number approaches zero, the α-shape develops cavities that could be converted in tunnels or holes. It expresses the intuitive notion of the shape of the point set and α is a parameter that controls the level of detail reflected by the polytope, that is to say, the maximum “curvature” of any cavity. As a result, the α-shape is homeomorphic to the original object sampled by the point cloud and approximates it within a fixed error bound.

As MLS only provides a partial view, the recovery of the non-visible side is achieved through symmetry, estimating the symmetry axis by a 180° field of view from the tree location. The hidden side of the canopy is closed by a flat surface. Finally, the canopy shape is obtained (Fig 3, step E) which allows the direct estimation of the CV.

#### Quality control

To validate and to assess the proposed methodology, different statistical evaluations are carried out (Fig 3, step F). The key parameter assessed is the DBH, since it is linked to various tree attributes. For DBH, the ground truth was acquired on location by tape in May 2016. The estimated accuracy of these measurements is reported as ±0.1 cm [39]. The protocol carried out in this field campaign was supported by [40]. The quality of the remaining parameters is assessed by TLS, measuring the trees in high-resolution from multiple directions to obtain complete and dense, high quality point clouds. This part of the field validation campaign was carried out in December 2016 (same phenotypic epoch as dataset 1) using a Leica Scan Station C10 TLS. The single point range accuracy of the Leica Scan Station C10 is reported to be 0.4 cm [41]. Between November 2015 and December 2016, no maintenance was done to these urban trees, guaranteeing similar tree parameters. A point cloud data expert estimated high quality parameter values from the TLS point clouds in a suitable graphical user interface.

The quality of the estimated parameters is expressed by the Squared Pearson Correlation Coefficient, or coefficient of determination (R^2^) and the Root Mean Square Error (RMSE) of the linear regression established between actual and estimated parameters. The Pearson correlation coefficient (R) only measures the precision of a linear relationship [42]. Also employed is Lin’s concordance correlation coefficient (CCC) [43] for assessing the agreement between quantitative measurements taken from different sources [44]. As result, it allows to evaluate reproducibility. At this point CCC (7) measures also the accuracy, yielding a value between -1 and 1, where 1 indicates a perfect agreement. It is computed as:

$$\text{CCC}=\frac{\text{2}\sigma_{\text{xy}}}{\sigma_{x}^{2}+\sigma_{y}^{2}+{\left(\mu_{\text{x}}-\mu_{\text{y}}\right)}^{\text{2}}}$$ (7)

where σ_xy_, σ_x_^2^, σ _y_^2^, μ_x_ and μ_x_ are the covariance, variances and means of the evaluated variables.

The RMSE provided by the regression is only valid if the sample follows a Gaussian distribution. So, the error sample is assessed by a Robust Jarque-Bera [45] normality test, implemented in the statistical software STAR (Statistics Tests for Analyzing of Residuals) [46]. In case the error sample does not follow the Gaussian hypothesis, the normalized median absolute deviation (NMAD) (8) is employed as a robust estimator of error dispersion.

NMAD=1.4826⋅MAD (8)

The NMAD allows to compare error dispersions from Gaussian samples, since it is normalized by the inverse of the cumulative distribution function of the Gaussian distribution (1.4826).

## Results

### Study area and field protocol

The study area is located at the Delft University of Technology campus (lat. 52°00′ N; long. 4°22′ E; 1 m a.s.l., The Netherlands) and tree species studied are *Aesculus hippocastanun* and *Quercus palustris*. Both species are deciduous and synoecious trees, widely cultivated in streets and parks throughout the temperate world. The acquisition was carried out at a medium speed of 40 km/h, with non-significant speed variation in the data adquisition. In order to get accurate and comparable results, only trees that are less than 10 meters from the road are considered. Two different datasets were analysed with distinct purposes; data referred to as dataset 1 provides validation of the methodology and dataset 2 analyses the scalability of the proposed methodology.

**Fig 4:**
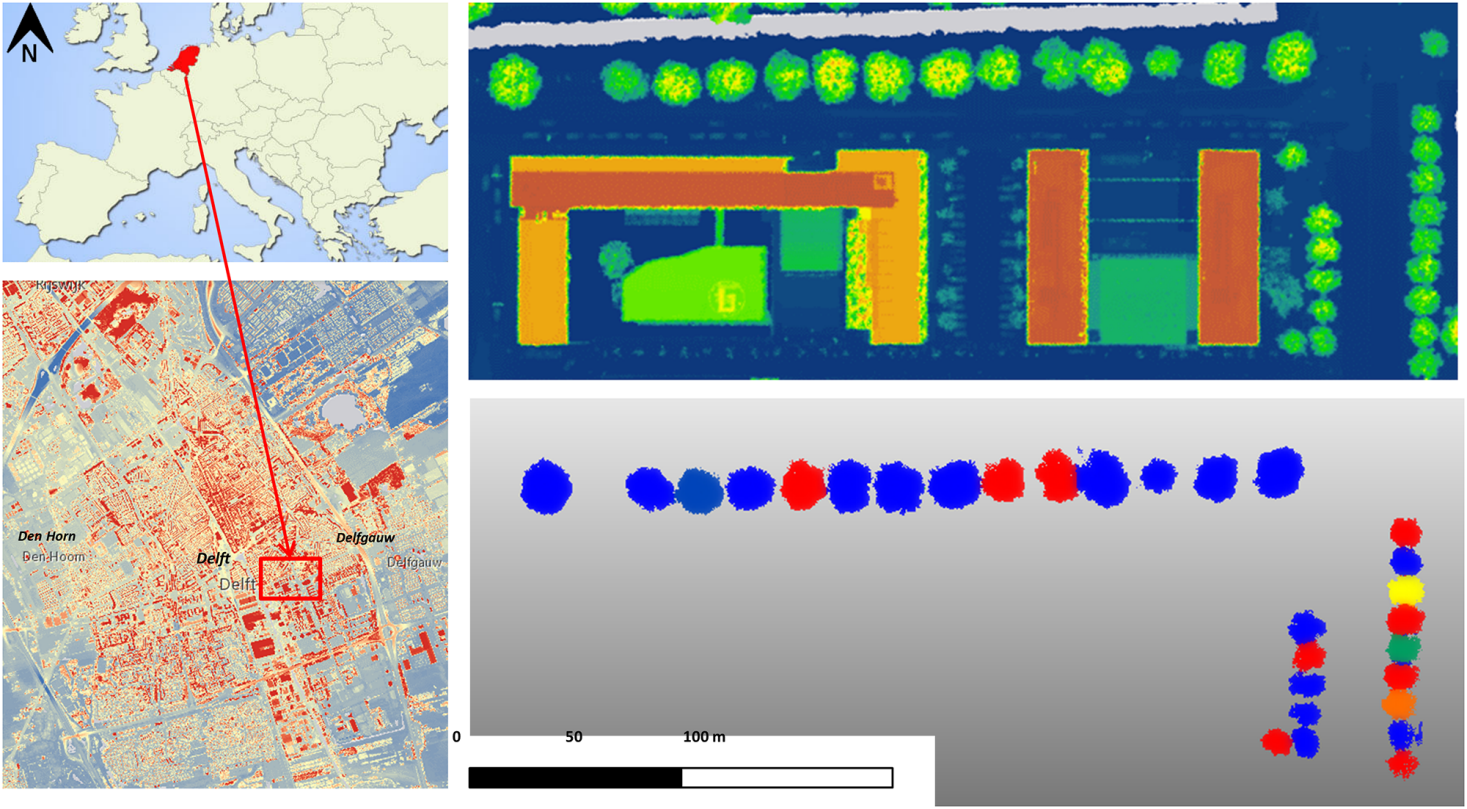
Study area (left) and dataset 1, view on floor (right): Aerial LiDAR (top) and point cloud from MLS of individual trees (bottom).

Dataset 1 (S1 Dataset) was collected on the 4^th^ of November 2015 covering an area of approximately 750 x 1200 m, on a day with a gentle breeze [47]. This dataset has 105,369,108 points and consists of *x*, *y*, *z* coordinates and laser intensity. A pre-processing phase of the dataset was carried out in order to obtain the individual trees of the zone of interest. After that, the point cloud has been reduced to 18,601,566 points, with an average amount of 640,000 points per tree. In the study zone are a total of 29 trees: 14 *Aesculus* and 15 *Quercus*, both in leaf-off condition. The first species are characterized by larger trunk diameters than the second. Fig 4 shows the location of the study area and dataset 1 outlined in red and the point clouds of the individual trees (in random colours) in plan-view, being the *Aesculus* horizontally placed and vertically the *Quercus*.

Dataset 2 was collected on the 5^th^ of May 2016 at the Delft University of Technology campus, covering an area of approximately 2100 x 3600 m, again on a day with a gentle breeze [47]. Following a similar approach, the final point cloud includes a total of 31,645,755 points for 58 *Aesculus hippocastanun* trees in leaf-on condition, which is equivalent to 560,000 points per tree on average.

### Experimental results

**Fig 5:**
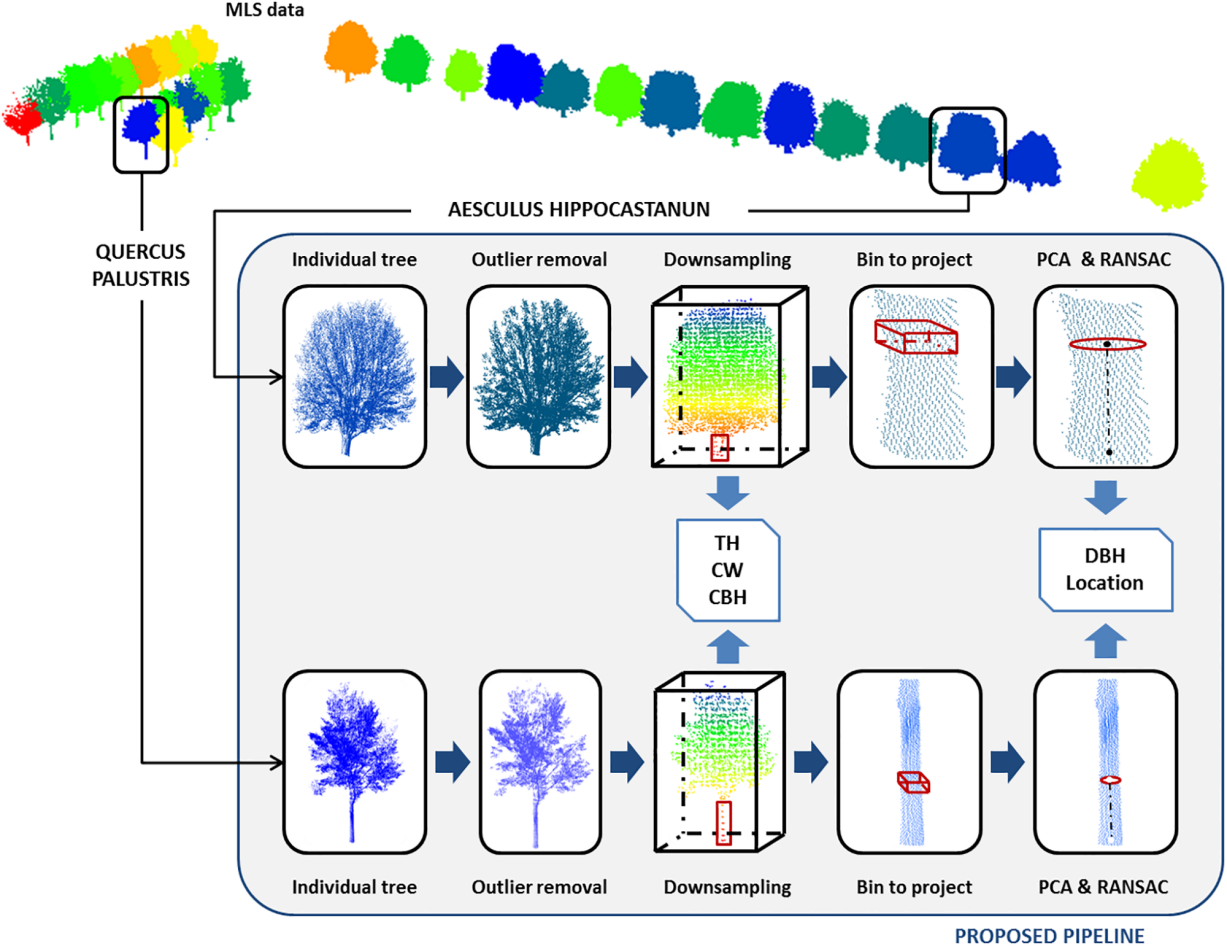
Schematic example of the proposed methodology according to species for dataset 1.

Both datasets were processed according to the proposed methodology and analysed correspondingly to the goal set: validation (dataset 1) and scalability (dataset 2). To demonstrate some characteristics of the MLS data, the point cloud density from dataset 1 is obtained. The point density of points sampling individual trunks is computed according to an ideal equilateral triangle distribution for a circular neighbourhood [48]. Table 2 shows the different density values, which depends on the tree species (see Fig 5 as example) and tree-MLS sensor distance: the higher density for the Quercus species corresponds to a lower tree-MLS system while the density variation is higher due to the tree morphology, as Table 2 shows.

**Table 2:** MLS point density for each tree.

Specie	Mean (cm)	Std (cm)
*Aesculus hippocastanun*	4.2	0.5
*Quercus palustris*	9.3	4.3

The different automatic processing steps required to obtain the structural tree parameters are summarized in Fig 5 for dataset 1.

The first step involves the determination of the CBH (Crown Base Height) to separate the canopy from the trunk by means of histogram analysis of the individual tree voxelization. In this case, a 10 cm voxel size is fixed to obtain CBH. For the determination of the other tree parameters, the original point cloud is employed, namely: TH (Tree Height), CW (Crown Width), DBH (Diameter at Breast Height), BCV (Base Crown Volume), CV (Canopy Volume) and location.

**Fig 6:**
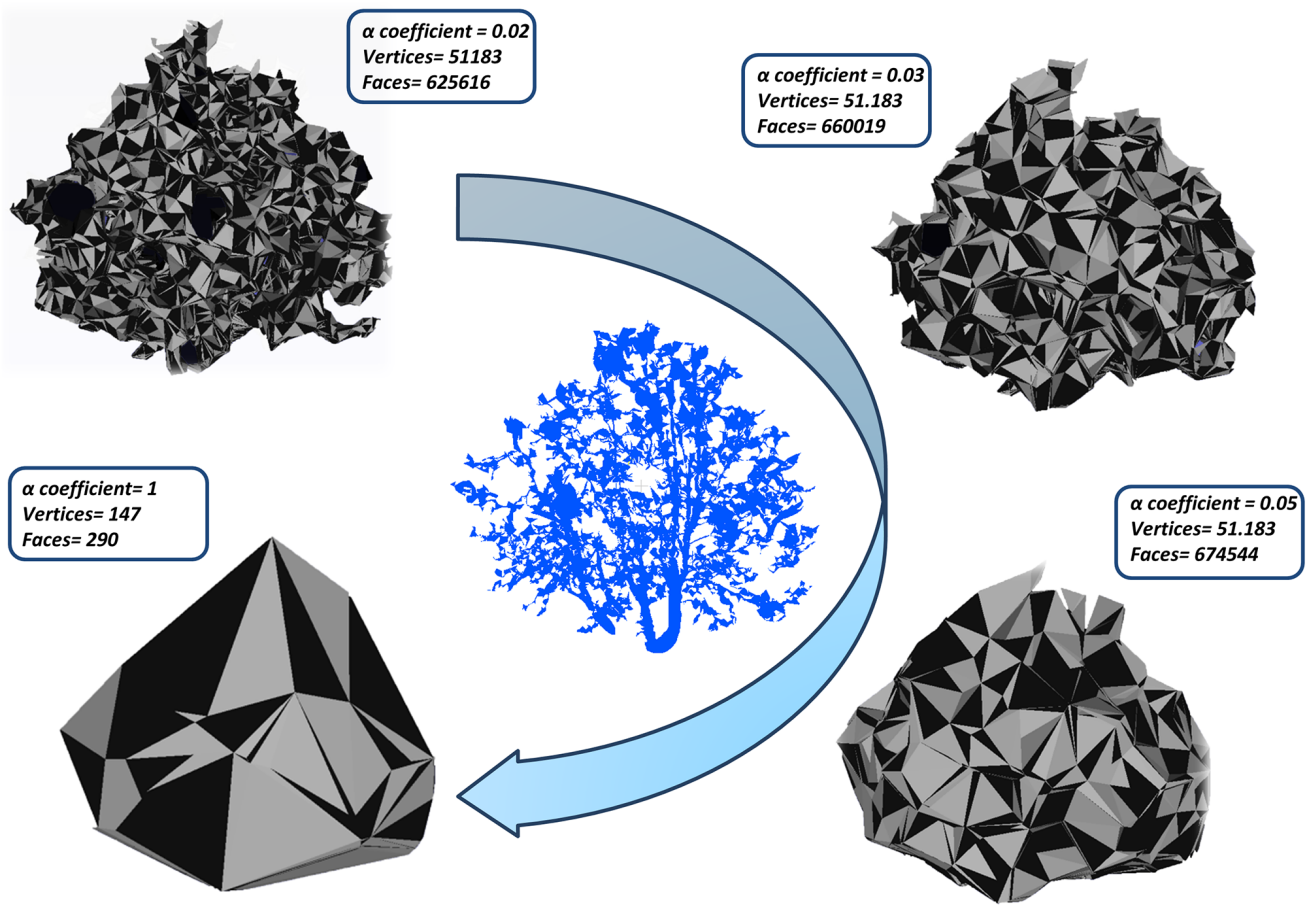
Canopy envelop results using different α coefficients from the triangulation mesh (the best result is obtained by α = 0.05).

In order to avoid any distortion in the methodology analysis, an outlier filter based on the so called Pope test [49] is applied to the discrepancies or residuals in DBH estimations. This outlier rejection test is based on the definition of standardized residuals, which are evaluated against a Tau distribution since they are not normally distributed. For a significance level of 5% the statistical threshold is set at 2.944, removing all points whose standardized residual is lower. The trees rejected by the Pope test have a non-circular trunk. Moreover, the BCV parameter derived directly from the stem curve (6) was computed according to the constraint that diameters from the ground plane until CBH are not larger than DBH. Altough the tree location is not a structural tree variable, it has special relevance in tree management in urban environments, compare for example [3]. Regarding volumetric measures, the α coefficient used to estimate the Canopy Volume (CV) parameter through the canopy envelop controls the level of detail of the final canopy. This effect is illustrated in Fig 6, where various canopy results are shown for different choices of the α coefficient. The volume variation in these cases is negligible (<3%). α = 0.05 is chosen, by a sensitive analysis, as the coefficient for estimating the canopy envelope. This value also complies with the homeomorphic requirements for shape-reconstruction and is the optimum one indicated by [50]. To tune this coefficient the the point cloud density and shape of the object should be taken into account. The main factor is the point density which is inverse correlated to the α coefficient, so for low spatial resolution datasets it should be increased. The secondary factor is the shape of the object as local shape variations affect local point density. If the separation distance between two branches is similar to the spatial resolution, they might be fused in the final envelope.

Final tree parameters from dataset 1, obtained by the proposed methodology following the specifications as shown in Table 3.

**Table 3:** Tree parameter results from dataset 1. Location coordinates (x UTM, y UTM), Tree Height (TH), Crown Width (CW), Diameter at Breast Height (DBH), Crown Base Height (CBH), Base Crown Volume (BCV) and Canopy Volume (CV).

ID	x _UTM_ (m)	y _UTM_ (m)	TH (m)	CW (m)	DBH (m)	CBH (m)	BCV (dm^3^)	CV (dm^3^)
1	85029.79	446503.46	15.21	13.35	0.75	1.4	85.2	898.9
2	85056.50	446514.77	13.80	13.51	0.67	1.3	46.5	726.8
3	85069.61	446520.18	16.21	12.82	0.67	1.3	58.1	880.3
4	85082.68	446526.32	15.00	12.84	0.99	1.4	100.2	809.5
5	85096.52	446532.46	13.72	12.63	0.76	1.3	59.4	662.7
6	85107.73	446537.74	16.01	12.42	1.08	1.8	119.4	1035.3
7	85119.77	446543.13	15.63	13.82	0.83	1.4	117.5	997.3
8	85134.52	446550.18	15.99	14.25	-	1.3	-	1168.9
9	85147.13	446555.82	15.31	11.7	0.77	1.3	68.4	777.1
10	85161.06	446562.13	13.91	13.76	0.50	2.2	44.2	621.4
11	85173.11	446567.45	16.99	14.45	0.77	1.2	95.7	1261.0
12	85187.02	446573.90	12.86	9.01	0.60	2.3	40.4	359.3
13	85201.51	446580.57	15.02	11.45	0.78	1.9	76.1	860.0
14	85218.22	446587.79	15.58	14.97	-	2.3	-	1042.1
15*	85257.16	446586.70	13.64	8.56	0.35	2.8	23.6	333.5
16*	85260.07	446579.31	14.17	7.59	0.32	3.2	18.6	333.4
17*	85263.18	446571.97	14.96	9.09	0.38	3.3	22.4	351.8
18*	85266.35	446564.60	14.60	8.59	0.55	2.7	46.7	355.6
19*	85269.26	446557.10	14.57	8.53	0.31	2.8	14.3	339.4
20*	85272.33	446549.97	15.31	7.34	0.40	3.3	22.6	300.7
21*	85275.20	446542.53	14.69	7.56	-	2.8	-	394.2
22*	85278.79	446534.73	14.27	7.38	0.34	3.7	24.1	347.3
23*	85281.45	446527.61	8.35	6.53	0.35	1.8	28.9	203.5
24*	85246.63	446518.54	12.84	7.22	0.40	2.9	24.2	252.7
25*	85253.94	446522.11	12.94	6.88	0.41	3.8	31.6	228.3
26*	85251.02	446529.52	14.00	6.41	0.36	4.3	33.3	210.6
27*	85247.81	446536.71	12.42	7.53	0.50	3.7	26.1	313.7
28*	85244.86	446544.16	16.10	7.53	0.37	2.8	17.6	407.7
29*	85241.91	446551.73	14.07	9.25	0.48	4.2	57.7	452.9

The *Quercus* trees are labelled with an asterisk (*).

Table 4 shows the average tree parameters of dataset 1 to highlight the different morphology of the trees involved in the experimental results.

**Table 4:** Tree summary from dataset 1.

Specie	TH (m)	CW (m)	DBH (m)	CBH (m)	BCV (dm^3^)	CV (dm^3^)
*Aesculus hippocastanun*	15.09	12.93	0.76	1.60	75.99	864.39
*Quercus palustris*	13.78	7.62	0.39	3.14	25.73	312.37

**Fig 7:**
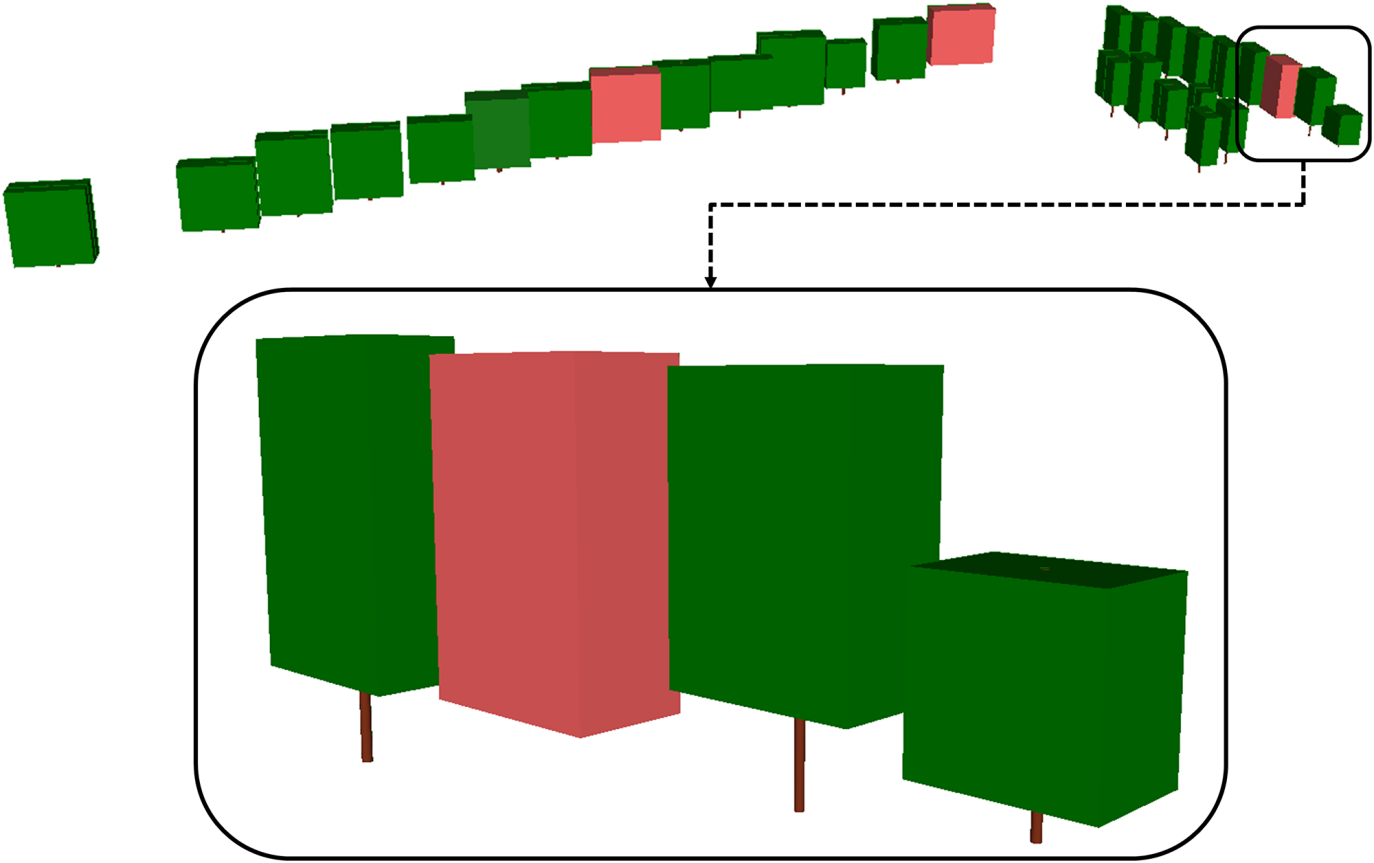
Tree parameter results from dataset 1. Schematic representation of tree location, TH, CW, DBH, BCH and CV.

One of the advantages of the availability of the tree parameters as given in Table 3, is the possibility to generate a simplified or structural, view of the individual trees without the need of employing the potentially many original MLS 3D points (Fig 6). This leads to a clear advantage in terms of quick visualization and data storage. This structural conceptualization shows the canopy as a rectangular prism such that its volume equals CV, its height the difference between TH and CBH and its width equals CW. Meanwhile, the trunk is simplified as a cylinder centred in the *x*,*y* coordinates, with a diameter equal to DBH and a height coincident to CBH. In case DBH was rejected by the Pope test, the canopy prism is marked in red in Fig 7 and the cylindrical trunk is omitted.

To evaluate the scalability at city level of the methodology, dataset 2 consisting of 58 individual *Aesculus hippocastanum* was processed. Table 5 sums up the average (μ) and standard deviation (σ) of the estimated parameters, providing an indication of the morphological homogeneity of the studied trees.

**Table 5:** Average (μ) and standard deviation (σ) of tree parameter results from dataset 2.

	TH (m)	CW (m)	DBH (cm)	CBH (m)	BCV (dm^3^)	CV (dm^3^)
μ	19.86	18.46	65.96	2.69	174.72	317.60
σ	1.09	2.70	13.85	1.13	64.49	58.40

**Fig 8:**
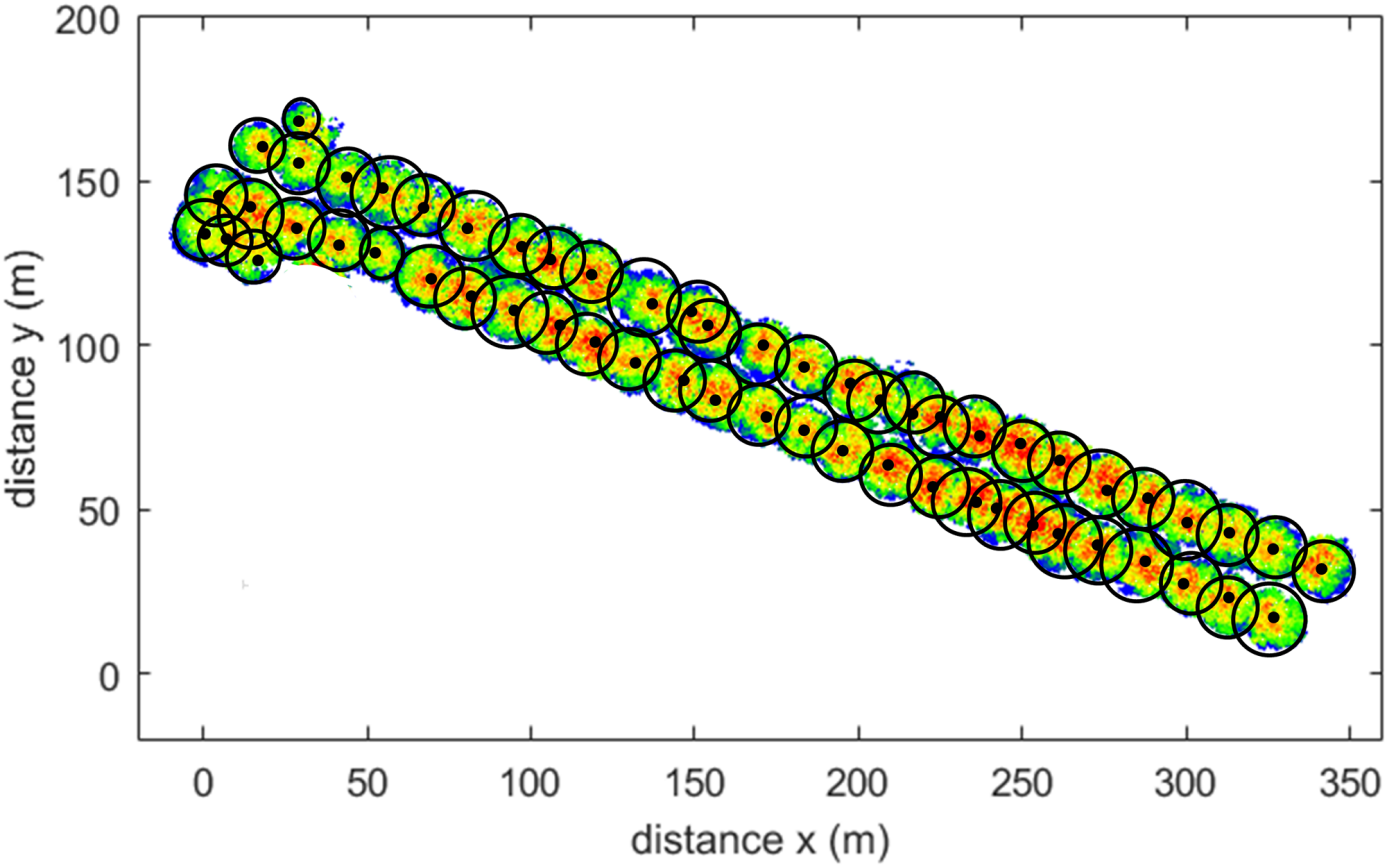
Tree parameter results (location and CW) overlapping to dataset 2 for scalability.

To highlight the scalability of the proposed methodology, in Fig 8, superimposed on the point cloud, are the CW values represented as a circumference centred in the computed *x*, *y* coordinates (black dot). This shows that the workflow is able to extract tree parameters even in high density tree areas, which is a common case in urban environments.

The computation time for dataset 2 is less than 10 seconds, indicating that the algorithm could be run at city level.

## Discussion

In this section, the sensitivity of the results with respect to the different parameters is discussed, as well as the validation of the results against *in situ* data.

### DBH analysis

**Fig 9:**
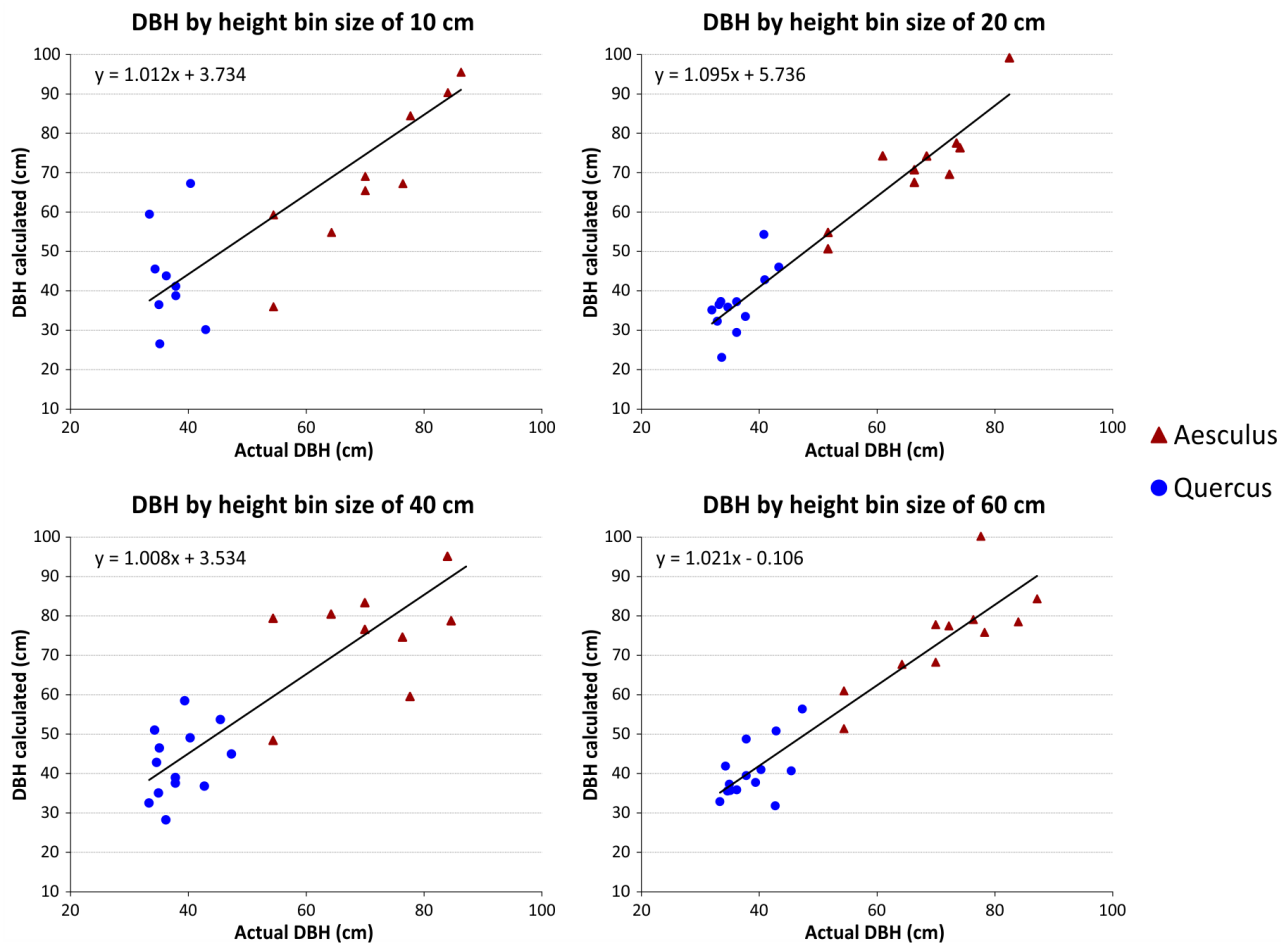
DBH linear regressions at different bin sizes for dataset 1.

First, the accuracy of the DBH estimation is evaluated for dataset 1. As commented in the previous section, different bin intervals along the trunk were considered to robustly fit DBH, namely: 1.25–1.35 m; 1.20–1.40 m; 1.10–1.50 m and 1.00–1.60 m. The linear regressions of DBH at different bin sizes are shown in Fig 9.

Linear regression parameters are listed in Table 6, being analysed the discrepancies between real and estimated DBH at different height bins. Both correlations coefficients: Pearson’s coefficient of determination (R^2^) as well as Lin’s Concordance Correlation Coefficient (CCC) are listed. The last one was computed as shown in (7). As comment, indicate that CCC cannot exceed the absolute value of Pearson’s correlation coefficient (R). For the error dispersion measurement, the sample is assessed by a robust Jarque-Bera normality test [45]. In all cases, the p-value of the normality test is higher than the significance level of 0.05, validating the RMSE as measure of error dispersion. Due to the nature of the applied regression, the error central tendency is compatible with zero.

**Table 6:** Summary of DBH determination for different heigh bins for dataset 1. Correlations coefficients (R^2^ and CCC), error dispersion (RMSE) and normality assessment of error sample.

Height Bin (m)	R^2^	CCC	RMSE (cm)	Normality (p-value)
**1.25–1.35**	0.69	0.80	10.7	0.63
**1.20–1.40**	0.92	0.95	4.9	0.87
**1.10–1.50**	0.76	0.85	9.2	0.42
**1.00–1.60**	0.89	0.94	6.0	0.84

According to the results in Table 5, we can conclude that the 20 cm height bin, using points from 1.20 m to 1.40 m height of each individual tree to estimate the DBH provides the best results.

Next, the influence of point cloud density on DBH estimation is analysed by considering the point density for different height bins and the corresponding error in DBH estimation. The results are listed in Table 7, where no significant correlation is shown. A bit higher correlation appears for the 20 cm interval results, but a check revealed that this is caused by the presence of an observation with a higher discrepancy, near to the outlier filter threshold, which was not automatically eliminated.

**Table 7:** Relationship between errors and MLS point density for each individual tree. Correlations coefficients (R^2^ and CCC).

Height Bin (m)	R^2^	CCC
**1.25–1.35**	0.0001	-0.0031
**1.20–1.40**	0.1873	0.1857
**1.10–1.50**	0.0023	0.0230
**1.00–1.60**	0.0019	-0.0191

Moreover, the number of points involved in the robust fitting process was compared against the regression parameters (Table 6). Table 8 lists the minimum, maximum and average number of points involved in the DBH fitting using different bin sizes. Additionally, the coefficients of determination among the different variables are shown, to illustrate their independency, namely: mean number of points for circle fitting versus RMSE, and versus CCC.

**Table 8:** Relationship between number of points and error in DBH estimation.

Height Bin (m)	Number of points			Coefficient of determination (R^2^)	
	Min	Mean	Max	RMSE	CCC
**1.25–1.35**	5	73.3	535	0.0494	0.0960
**1.20–1.40**	6	84.7	249		
**1.10–1.50**	11	245.6	2015		
**1.00–1.60**	17	346.9	2824		

### Analysis of other parameters

The rest of the tree parameters, Tree Height, Crown Width, BCV, BCV and Canopy volume are assessed in a similar way as the results presented in Table 5. In all cases both correlation coefficients are higher than 91% as shown in Table 9.

**Table 9:** Summary of tree parameters for dataset 1. Correlations coefficients (R^2^ and CCC), error dispersion (RMSE and NMAD) and normality assessment of error sample (p-value).

Parameter	R^2^	CCC	RMSE	NMAD	Normality (p-value)
**TH**	0.9801	0.9749	*(16*.*2 cm)*	5.9 cm	<0.0001
**CW**	0.9693	0.9770	*(47*.*0 cm)*	10.4 cm	<0.0001
**CBH**	0.9689	0.9771	15.7 cm	*(20*.*8 cm)*	0.3242
**BCV**	0.9186	0.9666	8.8 dm^3^	*(11*.*8 dm*^*3*^*)*	0.4687
**CV**	0.9970	0.9984	17.8 dm^3^	*(14*.*5 dm*^*3*^*)*	0.4464

Due to the non-normality of TH and CW, instead of RMSE, the NMAD (8) is employed as robust measure of error dispersion. Both values are listed in Table 9 for comparative purposes. In italics are shown that are rejected, according to the normality assessment. In the case of the TH and CW parameters, the RMSE tends to overestimate the error dispersion by a factor 2.8–4.5, as the error samples do not follow a normal distribution. So, the employment of RMSE as evaluator would lead to false the conclusions. For the other three parameters listed in Table 9, the normality is verified to be inside the 95% confidence level.

### Specifications of the study

**Fig 10:**
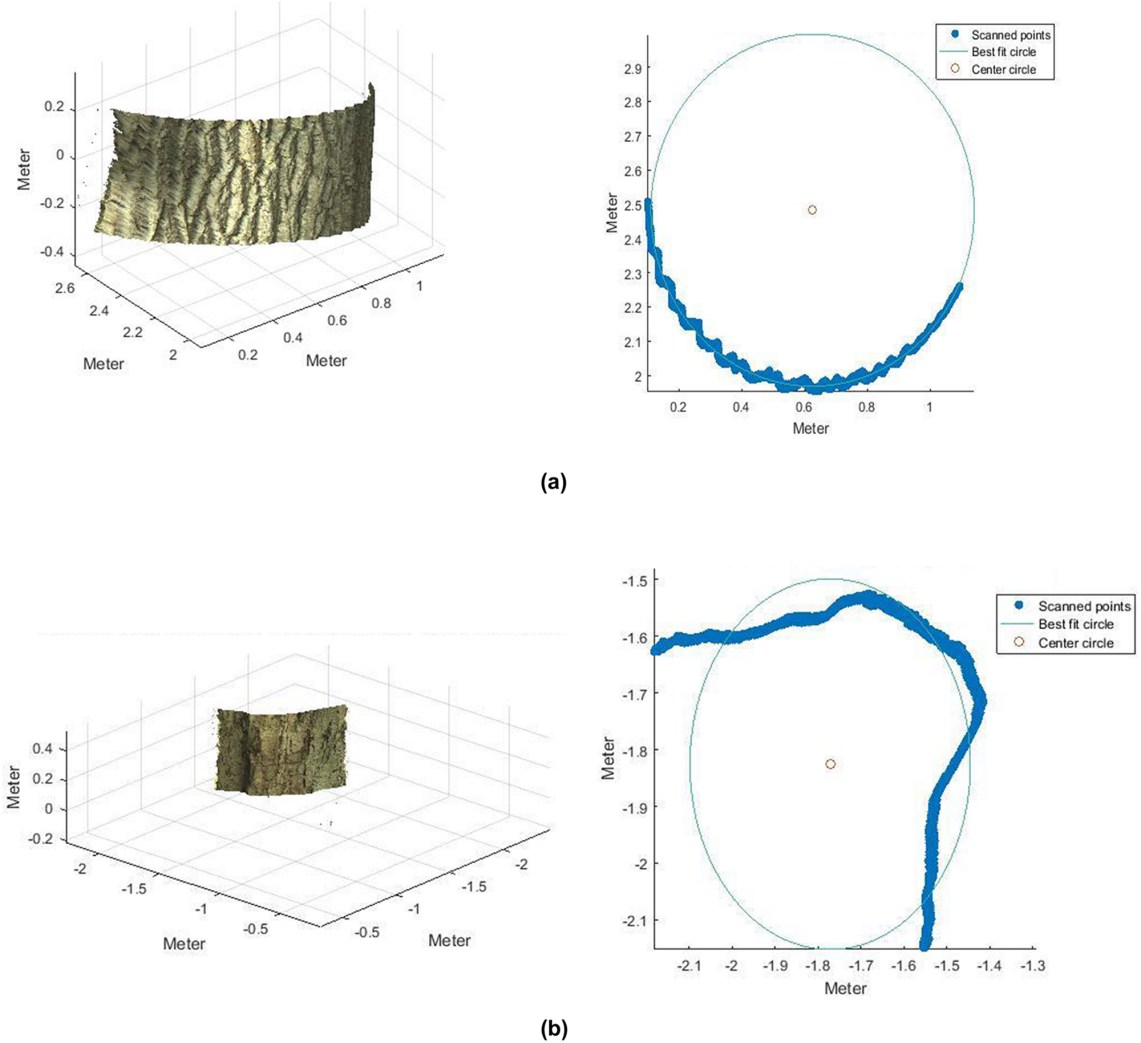
Trunk morphology effects on DBH estimation. Point cloud bin (left) and circle fit of the projected points in plan-view for a circular trunk shape (a) and a non-circular trunk shape (b). Both cases correspond to a 40 cm bin.

In some cases it was not possible to estimate DBH confidently due to the morphological shape of the tree trunk which caused the generation of a non-circular shape in the analysed bin. Fig 10 shows an example (Fig 10a) against a regular trunk shape (Fig 10b) of proposed methodology.

The geometric configuration of the sensor with respect to the trees did not result in underestimation for the height, width and canopy volume. By contrast, many studies in forest have reported tree height (TH) underestimation when attempting to use TLS for biomass estimation [21]. The cause is the complexity of the canopy obstructing the laser beam from reaching the top of the tree. In the present experiment, the laser was able to measure the full visible side of the urban trees.

**Fig 11:**
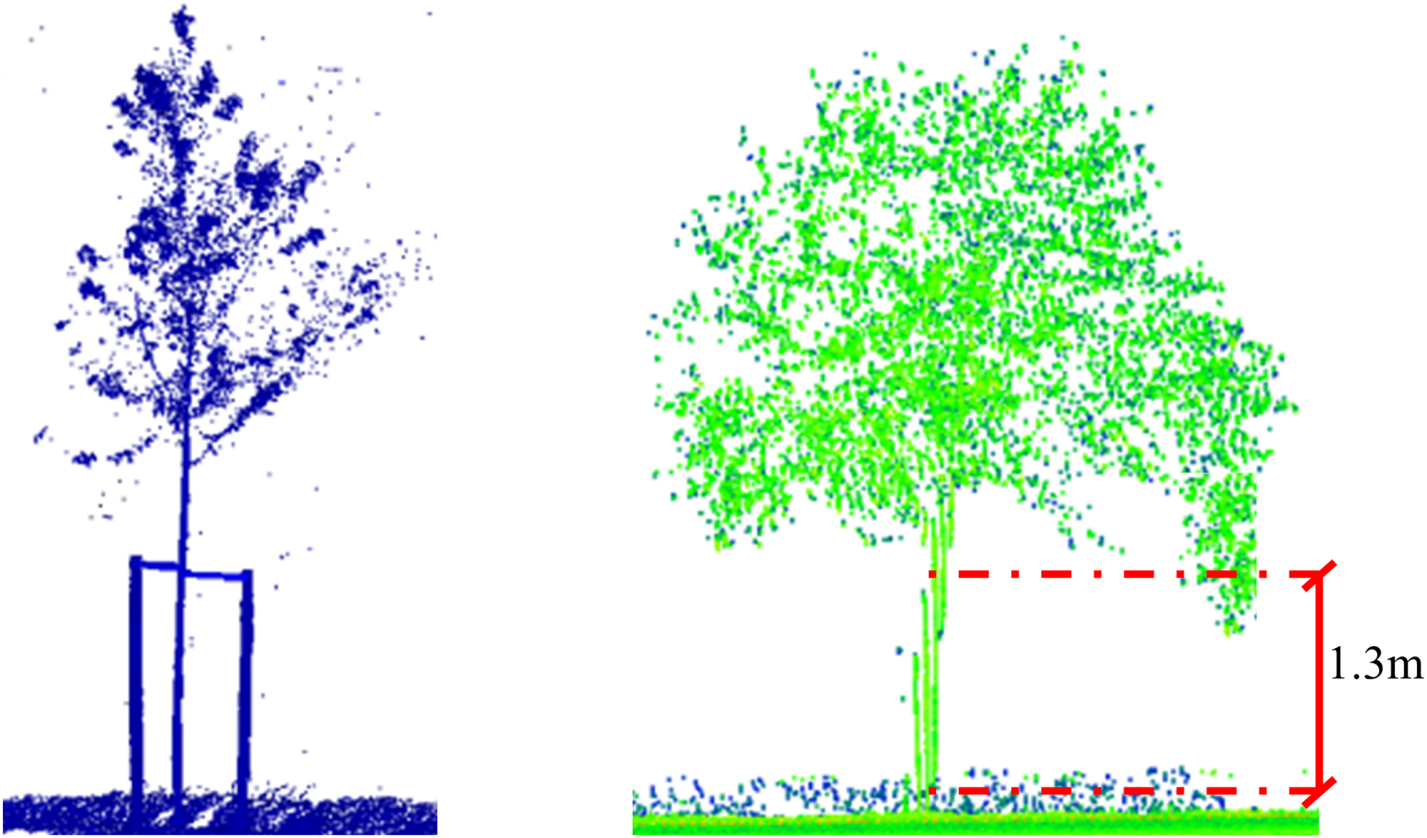
Examples of difficult tree scenarios.

However, the algorithm has to be improved for trunk detection, since in some scenarios it could derive erroneous DBH. These difficulties occur when there are objects in the trunk vicinity at a similar height, e.g. branches lower than CBH, or urban furniture/appliances in the trunk range (Fig 11). These examples are not present in the above datasets. In future studies, we will be focusing on providing geometric solutions to overcome this challenge. Another test will be dealing with the intensity value provided by the laser scanner to improve the segmentation between trunk and canopy.

A final comment is that the proposed methodology could be extrapolated to point clouds obtained by structure-from-motion technique. But in that case, results are expected to be affected by the inability to penetrate through the canopy, and the higher level of noise since it is a passive technique.

## Conclusions

The manuscript presents an efficient and non-invasive method for urban tree inventory based on point clouds acquired by MLS. The methodology is focused on the estimation of tree structural parameters in a fast, robust and objective way. Field measurements were used to validate these parameters. The methodology and derived results will help decision making related to urban trees.

This study discussed the influence of the number of points used for DBH estimation. Considering MLS point density and used height bin, no-significant relationship was reported. The optimal results in terms of correlation coefficient and error dispersion were for 20 cm bin heights, with a R^2^ of 0.92 and a RMSE of 4.9 cm. Moreover, the rest of the mentioned tree parameters were reported with a coefficient of determination higher than 0.91. The associated error was reported according to the best suitable statistis according to the error sample nature; RMSE or NMAD. In addition, the results show that working only on one side of the tree, the visible side from the road, is still feasible for approximating different tree structural parameters and canopy architecture with an accurate fit.

The methodology can be extrapolated to a comprehensive study of urban trees at city level [22]. Current experimental results indicate the reliability of the proposed algorithm and its possible employment when big data processing is required in an efficient way. The simple yet reliable approach makes the algorithm feasible for real-time processing and provides the advantage of running the workflow easily on different platforms.

Different applications from the proposed workflow would be broad-scale mapping, data fusion or calibration of remote sensing platforms to retrieve tree structural parameters and urban tree management. As a next work, we consider allometric relationships from the TIN to tree structure taking into account the point cloud density.

Future studies will address automated registration of LiDAR with imaging sensors, to satisfy the wide range of data requirements of urban tree characterization. The more promising sensors are multispectral and/or hyperspectral imaging sensors, which will allow a robust species classification.

## Supporting information

S1 Dataset Consists of a .xyz file of individual trees from MLS of dataset 1. (XYZ)
